# The lymphocyte-C-reactive protein ratio as the optimal inflammation-based score in patients with hepatocellular carcinoma underwent TACE

**DOI:** 10.18632/aging.202468

**Published:** 2021-02-11

**Authors:** Liang-He Lu, Wei Wei, Shao-Hua Li, Yong-Fa Zhang, Rong-Ping Guo

**Affiliations:** 1 Department of Hepatobiliary Oncology, Sun Yat-Sen University Cancer Center, State Key Laboratory of Oncology in South China, Collaborative Innovation Center for Cancer Medicine, Guangzhou 510060, China; 2 Department of Hepatic Surgery, Fudan University Shanghai Cancer Center, Shanghai 200032, China; 3 Department of Oncology, Shanghai Medical College, Fudan University, Shanghai 200032, China

**Keywords:** hepatocellular carcinoma, transarterial chemoembolization, inflammation-based score, lymphocyte-C-reactive protein ratio, prognosis

## Abstract

The lymphocyte-C-reactive protein ratio (LCR) is a recently described inflammation-based score, and it remains unclear which is the optimal inflammation-based score among patients with hepatocellular carcinoma (HCC) who underwent transarterial chemoembolization (TACE). A large cohort of HCC patients (n=1625) who underwent TACE as the initial treatment were enrolled in the present study. Inflammation-based scores, including the Glasgow Prognostic Score (GPS), modified Glasgow Prognostic Score (mGPS), high-sensitivity modified Glasgow Prognostic Score (Hs-mGPS), neutrophil-to-lymphocyte ratio (NLR), platelet-to-lymphocyte ratio (PLR), prognostic nutritional index (PNI), systemic immune-inflammation index (SII), and LCR, were all related to the survival of HCC patients, but only the LCR score was a significant and independent predictor in multivariate analysis (hazard ratio: 1.45; 95% confidence interval: 1.27-1.65; P<0.001). Further analysis showed that the LCR score stably and consistently differentiated subgroup patients with distinct prognoses. The predictive accuracies of the LCR score (0.70, 0.68, and 0.68 for 1-, 3-, and 5-year C-index, respectively) were superior to the other inflammatory-based scores (0.60-0.64, 0.58-0.62, and 0.58-0.62 for 1-, 3-, and 5-year C-index, respectively). The LCR score was an independent prognostic indicator for HCC patients who underwent TACE, and it was superior to the other inflammation-based scores in prognostic ability.

## INTRODUCTION

Hepatocellular carcinoma (HCC) is one of the most common devastating malignancies, and it is the leading cause of cancer-related mortality worldwide [1, 2]. Transarterial chemoembolization (TACE) is a widely used treatment modality because fewer than 20% of HCC patients are candidates for hepatic resection or local ablative therapy at the time of diagnosis [3]. However, the prognosis following TACE differs substantially, which is a major hurdle in optimizing HCC management strategies in clinical practice. The prognostic factors that influence survival should be extensively investigated to identify patients who will most likely benefit from TACE.

Cancer-related inflammation is the seventh hallmark of cancer [4]. The role of inflammatory markers in predicting oncological outcome was clearly evidenced with the development of numerous scoring systems: the Glasgow Prognostic Score (GPS) [5], modified Glasgow Prognostic Score (mGPS) [6], and the high-sensitivity modified Glasgow Prognostic Score (Hs-mGPS) [7], which measure serum C-reactive protein (CRP) and albumin levels; the neutrophil-to-lymphocyte ratio (NLR) [8]; the prognostic nutritional index (PNI) [9], which measures the serum albumin level and lymphocyte count; the platelet-to-lymphocyte ratio (PLR) [10]; and the systemic immune-inflammation index (SII) [11], which measures the neutrophil, lymphocyte and platelet counts. The lymphocyte-to-C-reactive protein ratio (LCR) was recently proposed as a simple discriminatory method in predicting of oncological outcomes [12–14]. This ratio is appealing due to its potential to categorize diverse prognostic subgroups, but the utility of the LCR score in HCC patients who underwent TACE was not well characterized. The identity of the components of the systemic inflammatory response that best predict cancer-specific survival for HCC patients who underwent TACE is not clear. Therefore, a reliable, easily accessible, and accurate risk prediction model to predict the ultimate survival outcomes of patients with HCC who underwent TACE must be identified and consolidated.

The present study evaluated the performance of the LCR score and performed a direct comparison of various inflammation-based scores in the predicting of overall survival (OS) for HCC patients who underwent TACE.

## RESULTS

### Patient characteristics

A total of 1625 patients were enrolled in this study. The baseline demographic and clinical characteristics are summarized in Supplementary Table 1. This cohort consisted of 1467 (90.3%) males and 158 (9.7%) females. A total of 884 (54.4%) patients were classified as albumin-bilirubin (ALBI) grade 1, and 741 (45.6%) patients were classified as ALBI grade 2. A total of 468 (28.8%) patients were divided into Barcelona Clinic Liver Cancer (BCLC) stage A, 642 (39.5%) patients into BCLC stage B, and 515 (31.7%) patients into BCLC stage C. A total of 905 (55.7%) patients had an LCR score >6000, and 720 (44.3%) patients had an LCR score ≤6000. The median OS time of the entire cohort was 15.7 (95% confidence interval (CI)): 14.3-16.8) months. At the time of censoring, 1168 of 1625 (71.9%) patients had died.

### Survival analysis

**Figure 1:**
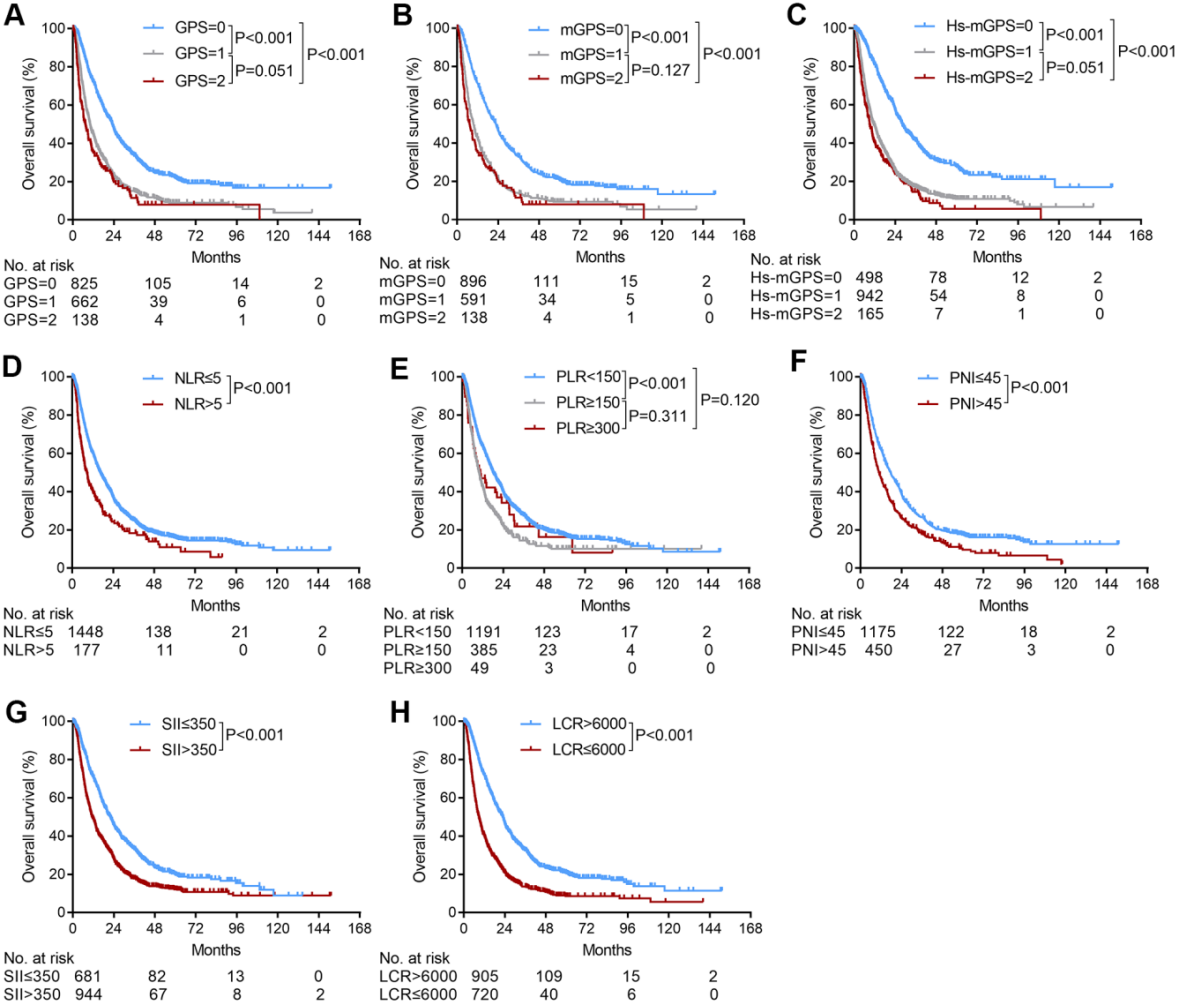
**The relationship between the inflammation-based scores and overall survival in patients with HCC who underwent TACE.** (**A**) GPS score, (**B**) mGPS score, (**C**) Hs-mGPS (**D**) NLR score, (**E**) PLR score, (**F**) PNI score, (**G**) SII score, (**H**) LCR score.

The Kaplan-Meier curves of the GPS, mGPS, Hs-mGPS, NLR, PLR, PNI, SII and LCR scores for the entire cohort are shown in Figure 1. Two groups with distinct survival curves were found for the LCR scores (1- and 3-year OS rates: 71.9% and 32.4% vs. 40.1% and 13.7%, P<0.001), the NLR scores (1- and 3-year OS rates: 60.1% and 25.0% v.s 40.6% and 18.3%, P<0.001), PNI scores (1- and 3-year OS rates: 62.1% and 26.7% vs. 47.2% and 17.9%, P<0.001), and the SII score (1- and 3-year OS rates: 70.6% and 33.2% vs. 48.9% and 17.9%, P<0.001). For the GPS, mGPS, Hs-mGPS, and PLR scores, no significant differences were observed between GPS 1 and GPS 2 (1- and 3-year OS rates: 44.6% and 15.1% vs. 35.1% and 11.5%, P=0.051), mGPS 1 and mGPS 2 (1- and 3-year OS rates: 42.6% and 14.0% vs. 35.1% and 11.5%, P=0.127), Hs-mGPS 1 and Hs-mGPS 2 (1- and 3-year OS rates: 49.1% and 17.2% vs. 40.3% and 14.5%, P=0.051), and PLR <150 and PLR≥300 (1- and 3-year OS rates: 63.5% and 27.6% vs. 47.0% and 21.8%, P=0.120), PLR ≥150 and PLR≥300 (1- and 3-year OS rates: 42.6% and 14.5% vs. 47.0% and 21.8%, P=0.311) (Figure 1).

### Risk factors for OS in HCC patients who underwent TACE

Univariate analysis revealed that albumin, total bilirubin, ALBI grade, alpha-fetoprotein (AFP), greatest tumor size, macroscopic vascular invasion, extrahepatic metastasis, BCLC stage, GPS score, mGPS score, Hs-mGPS, NLR score, PLR score, PNI score, SII score, and LCR score were associated with OS (Table 1). Multivariate analysis revealed that only the ALBI grade (2/1) (hazard ratio [HR] = 1.28, P < 0.001), AFP level (>/≤200 ng/ml) (HR=1.18, P=0.007), greatest tumor size (>/≤5 cm) (HR=1.56, P<0.001), BCLC stage (C/A, HR=2.15, P<0.001; B/A, HR=1.27, P=0.002), and LCR score (>/≤6000) (HR=1.45, P<0.001) were significant and independent factors for OS.

**Table 1:** Univariate and multivariate logistic regression analyses of overall survival.

**Variable**	**Univariate**		**Multivariate**	
	**HR (95%CI)**	**P**	**HR (95%CI)**	**P**
**Age (>/<60 years)**	0.91 (0.78-1.07)	0.256		
**Male/female**	1.03 (0.85-1.25)	0.738		
**Viral hepatitis**	0.91 (0.76-1.09)	0.319		
**ALBI grade (2/1)**	1.47 (1.31-1.65)	<0.001	1.28 (1.09-1.47)	<0.001
**AFP (>/<200 ng/ml)**	1.42 (1.26-1.59)	<0.001	1.18 (1.05-1.36)	0.007
**Greatest tumor size (>/<5 cm)**	2.21 (1.91-2.55)	<0.001	1.56 (1.33-1.83)	<0.001
**Tumor number (>/<1)**	1.06 (0.94-1.20)	0.318		
**Macroscopic vascular invasion (+/-)**	2.28 (2.01-2.59)	<0.001		
**Extrahepatic metastasis**	1.84 (1.55-2.18)	<0.001		
**BCLC stage**				
**A**				
**B**	1.30 (1.12-1.51)	0.001	1.27 (1.09-1.47)	0.002
**C**	2.74 (2.35-3.20)	<0.001	2.15 (1.84-2.52)	<0.001
**GPS score**				
**0**				
**1**	1.94 (1.71-2.19)	<0.001	1.33 (0.78-2.29)	0.296
**2**	2.38 (1.94-2.93)	<0.001	1.31 (0.66-2.58)	0.437
**mGPS score**				
**0**				
**1**	1.96 (1.74-2.22)	<0.001	0.82 (0.45-1.48)	0.503
**2**	2.32 (1.89-2.84)	<0.001		
**Hs-mGPS**				
**0**				
**1**	2.16 (1.89-2.48)	<0.001	1.41 (1.18-1.68)	0.056
**2**	2.60 (2.13-3.17)	<0.001	1.21 (0.67-2.21)	0.526
**NLR score (>/<5)**	1.53 (1.27-1.83)	<0.001	1.12 (0.92-1.38)	0.270
**PLR score**				
**0**				
**1**	1.58 (1.39-1.81)	<0.001	1.08 (0.92-1.26)	0.346
**2**	1.30 (0.93-1.82)	0.129	0.83 (0.576-1.20)	0.324
**PNI score (>/<45)**	1.43 (1.26-1.62)	<0.001	1.08 (0.90-1.29)	0.424
**SII score (>/<350)**	1.61 (1.43-1.81)	<0.001	0.97 (0.83-1.13)	0.717
**LCR score (>/<6000)**	2.05 (1.82-2.30)	<0.001	1.45 (1.27-1.65)	<0.001

Abbreviations: HR, hazard ratio; CI, confidence interval; ALBI grade, albumin-bilirubin grade; AFP, alpha-fetoprotein; BCLC stage, Barcelona Clinic Liver Cancer stage; GPS, Glasgow Prognostic Score; mGPS, modified Glasgow Prognostic Score; Hs-mGPS, the high-sensitivity modified Glasgow Prognostic Score; NLR, neutrophil-to-lymphocyte ratio; PLR, platelet-to-lymphocyte ratio; PNI, prognostic nutritional index; SII, systemic immune-inflammation index; LCR, lymphocyte-to-C-reactive protein ratio.

### Subgroup survival analysis

**Figure 2:**
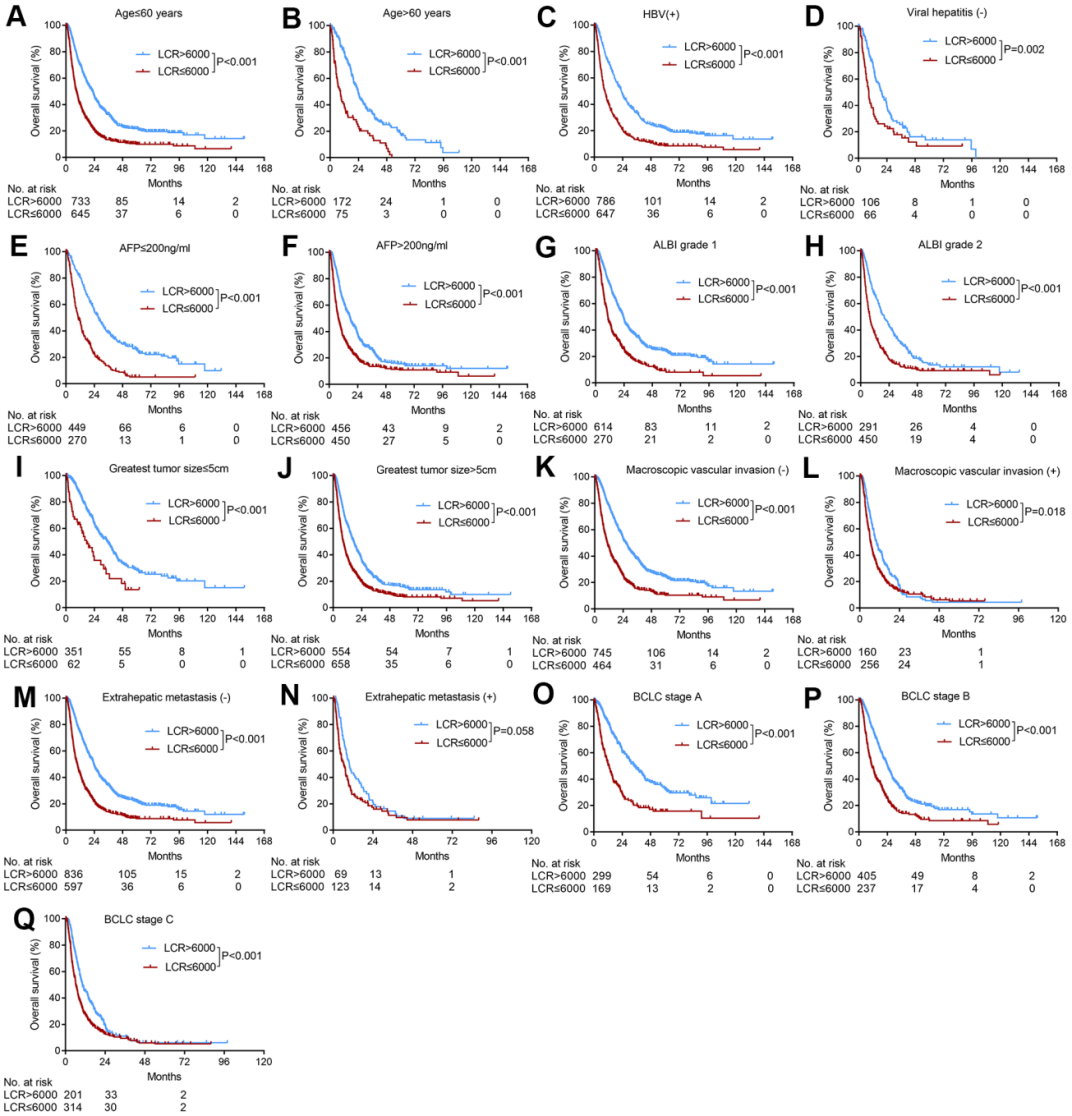
**Subgroup analyses of overall survival for HCC patients who underwent TACE according to various tumor characteristics.** (**A**) Age≤60 years; (**B**) age>60 years; (**C**) HBV positive; (**D**) absence of viral hepatitis (**E**) AFP≤200 ng/ml, (**F**) AFP>200 ng/ml, (**G**) ALBI grade 1, (**H**) ALBI grade 2, (**I**) greatest tumor size≤5 cm, (**J**) greatest tumor size>5 cm, (**K**) absence of macroscopic vascular invasion; (**L**) presence of macroscopic vascular invasion, (**M**) absence of extrahepatic metastasis; (**N**) presence of extrahepatic metastasis; (**O**) BCLC stage A, (**P**) BCLC stage B, (**Q**) BCLC stage C.

Based on the identified pretreatment predictors of OS in the multivariate Cox regression analyses, we further used the LCR score to subgroup patients based on age, etiology, AFP levels, ALBI grades, tumor sizes, macroscopic vascular invasion statuses, extrahepatic metastasis statuses, and BCLC stages. The LCR score stably and consistently divided these subgroups of patients into two groups with different prognoses (Figure 2), except for patients with extrahepatic metastasis (p=0.058) (Figure 2N).

### Comparison of the current commonly used inflammation-based prognostic systems

We used the concordance index (C-index) to identify which inflammation-based score performed best at predicting survival. The LCR score consistently had higher C-index values (0.70, 0.68, and 0.68 for the 1-, 3-, and 5-year C-indexes, respectively) than the other scores (0.60-0.63, 0.58-0.61, and 0.58-0.61 for the 1-, 3-, and 5-year C-indexes, respectively) for HCC patients who underwent TACE (Table 2).

**Table 2:** The concordance index used to compare different inflammation-based scores for HCC patients who underwent TACE.

**Inflammation-based score**	**C-index**					
	**1 year**	**P**	**3 years**	**P**	**5 yeas**	**P**
GPS	0.63	0.015	0.61	0.015	0.61	0.015
mGPS	0.63	0.015	0.61	0.015	0.61	0.015
Hs-mGPS	0.64	0.019	0.62	0.022	0.62	0.022
NLR	0.63	0.016	0.61	0.015	0.61	0.015
PLR	0.60	<0.001	0.59	<0.001	0.59	<0.001
PNI	0.60	<0.001	0.58	<0.001	0.58	<0.001
SII	0.62	0.010	0.61	0.015	0.60	0.010
LCR	0.70		0.68		0.68	

Abbreviations: GPS, Glasgow Prognostic Score; mGPS, modified Glasgow Prognostic Score; Hs-mGPS, the high-sensitivity modified Glasgow Prognostic Score; NLR, neutrophil-lymphocyte ratio; PLR, platelet lymphocyte ratio; PNI, prognostic nutritional index; SII, systemic immune-inflammation index; LCR, lymphocyte-to-C-reactive protein ratio.

*Statistical difference of C-index was investigated between the LCR score and various inflammatory-based scores.

## DISCUSSION

Increasing evidence has indicated that the systemic inflammatory response is a reliable predictor of cancer-specific survival, but the optimal response in HCC patients who undergo TACE is not clear. Our study used a large cohort and first showed that the recently described inflammation-based score, the LCR score, was stably and consistently discriminative in risk stratification across different subgroups of HCC patients who exhibited various liver function reserves and tumor characteristics.

Previous studies also reported that several inflammation-based scores emerged as predictors in the prognosis of patients with HCC who underwent TACE [15–22]. However, no study reported the optimal inflammation-based score in HCC patients who underwent TACE, and no study incorporated the LCR score. Our study found that the GPS, mGPS, Hs-mGPS, NLR, PLR, PNI, SII, and LCR scores were related to OS in univariate analysis, but only the LCR score (with the combination of lymphocytes and CRP, which are simple and objective but discriminatory in assessing high-risk HCC patients who underwent TACE) was the only independent predictor in the assessment of long-term survival in multivariate analysis. Therefore, measurement of the LCR score is an objective, easy to use, and simplified approach in clinical practice to facilitate the timely identification of patients who may benefit most from TACE or other treatment modalities.

Given the palliative intent of TACE, survival extent must be carefully weighed against treatment-related morbidity and mortality because TACE may worsen the underlying liver function, and it carries a potential risk of postprocedural mortality, which is as high as 10% [23]. The wide variation in OS after TACE is primarily because of the heterogeneous population of HCC patients with varying tumor burdens and liver functions. To maximize the efficacy of TACE, prognostic factors, including AFP level, tumor size, liver function reserve, and the presence of portal vein thrombosis [3, 24, 25], were extensively investigated to identify patients who were most likely to benefit from TACE, and these factors are consistent with the findings of our study. To extensively evaluate the flexibility of the LCR score, subgroup analyses in patients with various underlying liver functions and tumor burdens were performed, and our results demonstrated that the LCR score stably and consistently showed an outstanding distinguishing ability compared to the other inflammation-based scores.

The mechanism underlying the association between the LCR score and long-term outcome for HCC patients who underwent TACE was not clarified. It is widely acknowledged that the growth and metastasis of tumors result from complex interactions between tumors and stromal factors [26], including blood vessels, inflammatory cells and the immune system, which lead to chronic inflammation. HCC is a typical inflammation-driven disease that primarily develops due to underlying chronic liver inflammation, and sustained inflammation may reflect a pro-angiogenic tumor microenvironment. For the LCR score, the lymphocyte count plays a critical role in the host cytotoxic immune response to tumors, and it likely reflects the generalized state of immune function that results in the release of various cytokines and inhibits the growth of cancer cells [27]. Serum CRP plays a pivotal role in tumor development. Elevated CRP levels are a good indicator of T-lymphocyte impairment [28, 29], which is associated with circulating concentrations of vascular endothelial growth factors [30]. Therefore, the outstanding potential of the LCR score to distinguish diverse prognostic subgroups was conceivable with the combination of lymphocytes and CRP measurements.

There are several limitations in this study. First, due to the retrospective nature of the analysis, deviation from the clinical design was unavoidable. Second, this study was performed at a single-center with hepatitis B virus (HBV) (88.2%) as the main risk factor for HCC, and the results need further validation in non-HBV-predominant populations. Third, the capacity of the LCR score for HCC patients with macroscopic vascular invasion or extrahepatic metastasis was limited, and the molecular alterations underlying inflammation in HCC patients who underwent TACE are not completely understood and need further basic study.

In summary, our study demonstrated that the LCR score showed a stable and consistent differentiation capacity in HCC patients who underwent TACE. The LCR score is a promising marker in the management of HCC patients who undergo TACE.

## MATERIALS AND METHODS

### Study population

A total of 1625 HCC patients who underwent TACE as the initial treatment at the Sun Yat-Sen University Cancer Center from October 2006 to August 2014 were enrolled. The following key eligibility criteria were used: a) aged between 18 and 75 years; b) HCC confirmed by radiological or histological evaluation based on the American Association for the Study of Liver Diseases or European Association for the Study of the Liver guidelines [31]; c) patients who underwent TACE as the initial treatment; and d) Eastern Cooperative Oncology Group performance scores ≤1. The following exclusion criteria were used: a) evidence of hepatic decompensation, including ascites, esophageal or gastric variceal bleeding or hepatic encephalopathy; b) patients who underwent TACE as a bridging treatment prior to liver transplantation; and c) the presence of an additional primary malignancy in another organ.

All clinicopathological variables, including demographics, full blood counts, albumin, total bilirubin, CRP, AFP, ALBI grade [32], tumor burden (including greatest tumor size, tumor number, macroscopic vascular invasion, and extrahepatic metastasis), BCLC stage, and inflammation-based scores (including the GPS, mGPS, Hs-mGPS, NLR, PLR, PNI, SII, and LCR scores), were collected at the time of referral to our unit prior to treatment.

The protocol was performed according to the standards of the Helsinki Declaration and was approved by the Sun Yat-Sen University Cancer Center Ethics Committees. Written informed consent was obtained from all patients.

### TACE procedure

TACE was performed using previously described techniques [33, 34]. Before the TACE procedure, a selective 5-F catheter was introduced, and visceral angiography was performed to assess portal vein patency, vascular anatomy, and tumor vascularity. Depending on the arterial supply of the tumor identified by arteriography, a 2.7 Fr microcatheter (Terumo Corporation, Tokyo, Japan) was superselectively placed into the feeding arteries of the tumor and the tumor thrombus for selective embolization, which was performed using an embolization suspension consisting of 6 mg mitomycin C (Zhejiang Hisun Pharmaceutical, Taizhou, China), 50 mg lobaplatin (Bristol-Myers Squibb, New York, NY), and 50 mg epirubicin (Pharmorubicin; Pfizer, Wuxi, China) mixed with Lipiodol (Lipiodol; Guerbet, Villepinte, France). For all patients, embolization was finally performed with absorbable gelatin-sponge particles (Gelfoam; Hanzhou Alc, Hangzhou, China; 1 to 2 mm in diameter).

### Follow-up

Follow-up examinations included laboratory tests (e.g., serum AFP, liver function, and blood tests), abdominal contrast-enhanced computer tomography (CT) [14] or magnetic resonance imaging (MRI). All patients visited the clinic 4–6 weeks after each cycle of TACE. TACE was repeated on demand if residual viable tumor tissue was evident on sequential dynamic liver CT without deterioration of hepatic function. The study was censored on December 31, 2019.

### Diagnosis and definitions

The inflammation-based scores, including GPS, mGPS, Hs-mGPS, NLR, PLR, PNI, SII and LCR, were calculated from existing preoperative laboratory parameters using the equation shown in Table 3. The cut-off values of inflammatory-based scores in this study were determined as previous studies reported [5–10, 12–14]. All patients enrolled in this study underwent blood testing at Sun Yat-Sen University Cancer Center. Measurements of all inflammatory-based scores were performed within 7 days before TACE.

**Table 3:** Inflammation-based prognostic scoring systems.

**Scoring systems**	**Score**
**The Glasgow Prognostic** **Score**	
CRP (≤10 mg/L) and albumin (≥35 g/L)	0
CRP (≤10 mg/L) and albumin (<35 g/L)	1
CRP (>10 mg/L) and albumin (≥35 g/L)	1
CRP (>10 mg/L) and albumin (<35 g/L)	2
**The modified Glasgow Prognostic Score**	
CRP (≤10 mg/L) and albumin (≥35 g/L)	0
CRP (≤10 mg/L) and albumin (<35 g/L)	0
CRP (>10 mg/L) and albumin (≥35 g/L)	1
CRP (>10 mg/L) and albumin (<35 g/L)	2
**The high-sensitivity modified Glasgow Prognostic Score**	
CRP (≤3 mg/L) and albumin (≥35 g/L)	0
CRP (≤3 mg/L) and albumin (<35 g/L)	0
CRP (>3 mg/L) and albumin (≥35 g/L)	1
CRP (>3 mg/L) and albumin (<35 g/L)	2
**Neutrophil-to-lymphocyte ratio**	
Neutrophil count: lymphocyte count ≤5:1	0
Neutrophil count: lymphocyte count >5:1	1
**Platelet-to-lymphocyte ratio**	
Platelet count: lymphocyte count <150:1	0
Platelet count: lymphocyte count ≥150:1	1
Platelet count: lymphocyte count >300:1	2
**Prognostic nutritional index**	
Albumin (g/L) +5× total lymphocyte count (10^9^/L) >45	0
Albumin (g/L) +5× total lymphocyte count (10^9^/L) ≤45	1
**Systemic immune-inflammation index (SII)**	
Platelet count (×10^9^/L) × neutrophil count (×10^9^/L)/lymphocyte count (×10^9^/L) ≤350	0
Platelet count (×10^9^/L) × neutrophil count (×10^9^/L)/lymphocyte count (×10^9^/L) >350	1
**Lymphocyte-to-C-reactive protein ratio**	
10^4^× lymphocyte count (10^9^/L): CRP (mg/L) >6000	0
10^4^× lymphocyte count (10^9^/L): CRP (mg/L) ≤6000	1

Abbreviation: CRP: C-reactive protein.

### Statistical analysis

The primary endpoint was OS, which was calculated as the interval between the date of TACE until the date of death or the last follow-up. Survival time was estimated using the Kaplan-Meier method, and the survival difference between groups was assessed using the log-rank test. The inflammation-based scores, including the GPS, mGPS, Hs-mGPS, NLR, PLR, PNI, SII, and LCR scores, were calculated as described previously. All variables that were significant in univariate analysis were included in the Cox multivariate analysis. The C-index method was calculated by R v3.6.1 (www.r-project.org), and used to rank the different inflammatory-based scores according to their capacity to discriminate patients. All tests were two-sided, and P < 0.05 was considered significant. Data analyses were performed using SPSS 25.0 (SPSS Inc., Chicago, IL, USA), and R v3.6.1 (www.r-project.org).

## Supplementary Material

Supplementary Table 1
