# Supplementary material for: The lymphocyte-C-reactive protein ratio as the optimal inflammation-based score in patients with hepatocellular carcinoma underwent TACE
Source: Aging (Albany NY). 2021 Feb 11;13(4):5358–68. doi: 10.18632/aging.202468 (PMC7950222; doi:10.18632/aging.202468)
Supplement: Supplementary Table 1 [file aging-13-202468-s001.docx]

| **Supplementary Table 1. Baseline characteristics of subgroups divided by various inflammation-based scores.** | **All patient (n=1625)** | **GPS** | | | **P** | **mGPS** | | | **P** | **Hs-mGPS** | | | **P** | **NLR** | | **P** | **PLR** | | | **P** | **PNI** | | **P** | **SII** | | **P** | **LCR** | | **P** |
| --- | --- | --- | --- | --- | --- | --- | --- | --- | --- | --- | --- | --- | --- | --- | --- | --- | --- | --- | --- | --- | --- | --- | --- | --- | --- | --- | --- | --- | --- |
|  |  | **0** | **1** | **2** |  | **0** | **1** | **2** |  | **0** | **1** | **2** |  | **≤5** | **>5** |  | **<150** | **≥150** | **≥300** |  | **≤45** | **>45** |  | **≤350** | **>350** |  | **≤6000** | **>6000** |  |
| **Age (year)** |  |  |  |  | <0.001 |  |  |  | <0.001 |  |  |  | <0.001 |  |  | 0.277 |  |  |  | <0.001 |  |  | 0.308 |  |  | <0.001 |  |  | <0.001 |
| ≤60 | 1378  (84.8) | 669  (41.1) | 586  (36.1) | 123  (7.6) |  | 723  (44.5) | 532  (32.7) | 123  (7.6) |  | 395  (24.3) | 826  (50.8) | 157  (9.7) |  | 1223  (75.3) | 155  (9.5) |  | 980  (60.3) | 352  (21.7) | 46  (2.8) |  | 1003  (61.7) | 375  (23.1) |  | 544  (33.5) | 834  (51.3) |  | 733  (45.1) | 645 (39.7) |  |
| >60 | 247  (15.2) | 156  (9.6) | 76  (4.7) | 15  (0.9) |  | 173  (10.6) | 59  (3.6) | 15  (0.9) |  | 103  (6.3) | 116  (7.1) | 28  (1.7) |  | 225  (13.8) | 22  (1.4) |  | 211  (13.0) | 33  (2.0) | 3  (0.2) |  | 172  (10.6) | 75  (4.6) |  | 137  (8.4) | 110  (6.8) |  | 172  (10.6) | 75  (4.6) |  |
| **Gender** |  |  |  |  | 0.145 |  |  |  | 0.131 |  |  |  | 0.141 |  |  | 0.630 |  |  |  | 0.001 |  |  | 0.123 |  |  | 0.475 |  |  | 0.311 |
| Male | 1467 (90.3) | 734  (45.2) | 609  (37.5) | 124  (7.6) |  | 798  (49.1) | 545  (33.5) | 124  (7.6) |  | 439  (27.0) | 861  (53.0) | 167  (10.3) |  | 1309  (80.6) | 158  (9.7) |  | 1095  (67.4) | 332  (20.4) | 40  (2.5) |  | 1069  (65.8) | 398  (24.5) |  | 619  (38.1) | 848  (52.2) |  | 811  (49.9) | 656 (40.4) |  |
| Female | 158  (9.7) | 91  (5.6) | 53  (3.3) | 14  (0.9) |  | 98  (6.0) | 46  (2.8) | 14  (0.9) |  | 59  (3.6) | 81  (5.0) | 18  (1.1) |  | 139  (8.6) | 19  (1.2) |  | 96  (5.9) | 53  (3.3) | 9  (0.6) |  | 106  (6.5) | 52  (3.2) |  | 62  (3.8) | 96  (5.9) |  | 94 (5.8) | 64  (3.9) |  |
| **Etiology** |  |  |  |  | 0.324 |  |  |  | 0.030 |  |  |  | 0.074 |  |  | 0.807 |  |  |  | 0.001 |  |  | <0.001 |  |  | <0.001 |  |  | 0.022 |
| HBV | 1433 (88.2) | 718  (44.2) | 590  (36.3) | 125  (7.7) |  | 781  (48.1) | 527  (32.4) | 125  (7.7) |  | 431  (26.5) | 834  (51.3) | 168  (10.3) |  | 1283  (79.0) | 150  (9.2) |  | 1059  (65.2) | 340  (20.9) | 34  (2.1) |  | 1021  (62.8) | 412  (25.4) |  | 616  (37.9) | 817  (50.3) |  | 786  (48.4) | 647 (39.8) |  |
| HCV | 20  (1.2) | 11  (0.7) | 7  (0.4) | 2  (0.1) |  | 16  (1.0) | 2  (0.1) | 2  (0.1) |  | 10  (0.6) | 7  (0.4) | 3  (0.2) |  | 17  (1.0) | 3  (0.2) |  | 18  (1.1) | 0  (0.0) | 2  (0.1) |  | 11  (0.7) | 9  (0.5) |  | 16  (1.0) | 4  (0.2) |  | 16 (1.0) | 4  (0.2) |  |
| Others | 172  (10.6) | 98  (6.0) | 65  (4.0) | 9  (0.6) |  | 102  (6.3) | 61  (3.7) | 9  (0.6) |  | 57  (3.5) | 103  (6.4) | 12  (0.7) |  | 153  (9.4) | 19  (1.2) |  | 118  (7.3) | 44  (2.7) | 10  (0.6) |  | 145  (8.9) | 27  (1.7) |  | 52  (3.2) | 120  (7.5) |  | 106 (6.5) | 66  (4.1) |  |
| **ALBI grade** |  |  |  |  | <0.001 |  |  |  | <0.001 |  |  |  | <0.001 |  |  | <0.001 |  |  |  | <0.001 |  |  | <0.001 |  |  | 0.336 |  |  | <0.001 |
| 1 | 884  (54.4) | 599  (36.9) | 285  (17.5) | 0  (0.0) |  | 599  (36.9) | 285  (17.5) | 0  (0.0) |  | 354  (21.8) | 530  (32.6) | 0  (0.0) |  | 824  (50.7) | 60  (3.7) |  | 682  (42.0) | 184  (11.3) | 18  (1.1) |  | 853  (52.5) | 31  (1.9) |  | 380  (23.4) | 50  (31.0) |  | 614 (37.8) | 270  (16.6) |  |
| 2 | 741  (45.6) | 226  (13.9) | 377  (23.2) | 138  (8.5) |  | 297  (18.3) | 306  (18.8) | 138  (8.5) |  | 144  (8.9) | 412  (25.4) | 185  (11.4) |  | 624  (38.4) | 117  (7.2) |  | 509  (31.3) | 201  (12.4) | 31  (1.9) |  | 322  (19.8) | 419  (25.8) |  | 301  (18.5) | 440  (27.1) |  | 291 (17.9) | 450 (27.7) |  |
| **Alpha-fetoprotein level (ng/L)** |  |  |  |  | <0.001 |  |  |  | <0.001 |  |  |  | <0.001 |  |  | 0.710 |  |  |  | 0.001 |  |  | 0.365 |  |  | <0.001 |  |  | <0.001 |
| ≤200 | 719  (44.2) | 404  (24.9) | 263  (16.2) | 52  (3.2) |  | 442  (27.2) | 225  (13.8) | 52  (3.2) |  | 261  (16.1) | 380  (23.4) | 78  (4.8) |  | 643  (39.6) | 76  (4.7) |  | 559  (34.4) | 142  (8.7) | 18  (1.1) |  | 528  (32.5) | 191  (11.8) |  | 346  (21.3) | 373  (23.0) |  | 449 (27.6) | 270 (16.6) |  |
| >200 | 906  (55.8) | 421 (25.9) | 399  (24.6) | 86  (5.3) |  | 454  (27.9) | 366  (22.5) | 86  (5.3) |  | 237  (14.6) | 562  (34.6) | 107  (6.6) |  | 805  (49.5) | 101  (6.2) |  | 632  (38.9) | 243  (15.9) | 31  (1.9) |  | 647  (39.8) | 259  (15.9) |  | 335  (20.6) | 571  (35.1) |  | 456 (28.1) | 450  (27.7) |  |
| **Tumor size (cm)** |  |  |  |  | <0.001 |  |  |  | <0.001 |  |  |  | <0.001 |  |  | <0.001 |  |  |  | <0.001 |  |  | 0.763 |  |  | <0.001 |  |  | <0.001 |
| ≤5 | 413  (25.4) | 319  (19.6) | 80  (4.9) | 14  (0.9) |  | 354  (21.8) | 45  (2.8) | 14  (0.9) |  | 260  (16.0) | 119  (7.3) | 34  (2.1) |  | 394  (24.2) | 19  (1.2) |  | 389  (23.9) | 23  (1.4) | 1  (0.1) |  | 301  (18.5) | 112  (6.9) |  | 317  (19.5) | 96  (5.9) |  | 351 (21.6) | 62  (3.8) |  |
| >5 | 1212  (74.6) | 506  (31.1) | 582  (35.8) | 124  (7.6) |  | 542  (33.4) | 546  (33.6) | 124  (7.6) |  | 238  (14.6) | 823  (50.6) | 151  (9.3) |  | 1054  (64.9) | 158  (9.7) |  | 802  (49.4) | 362  (22.3) | 48  (3.0) |  | 874  (53.8) | 338  (20.8) |  | 364  (22.4) | 848  (52.5) |  | 554 (34.1) | 658 (40.5) |  |
| **Tumor number** |  |  |  |  | 0.029 |  |  |  | 0.031 |  |  |  | 0.117 |  |  | 0.013 |  |  |  | 0.004 |  |  | 0.011 |  |  | 0.021 |  |  | 0.059 |
| ≤1 | 631  (38.8) | 316  (19.4) | 247  (15.2) | 68  (4.2) |  | 340  (20.9) | 223  (13.7) | 68  (4.2) |  | 183  (11.3) | 364  (22.4) | 84  (5.2) |  | 547  (33.7) | 84  (5.2) |  | 435  (26.8) | 177  (10.9) | 19  (1.2) |  | 434  (26.7) | 197  (12.1) |  | 242  (14.9) | 389  (23.9) |  | 333 (20.5) | 298  (18.3) |  |
| >1 | 994  (61.2) | 509 (31.3) | 415  (25.5) | 70  (4.3) |  | 556  (34.2) | 368  (22.6) | 70  (4.3) |  | 315  (19.4) | 578  (35.6) | 101  (6.2) |  | 901  (55.4) | 93  (5.7) |  | 756  (46.5) | 208  (12.8) | 30  (1.8) |  | 741  (45.6) | 253  (15.6) |  | 439  (27.0) | 555  (34.2) |  | 572 (35.2) | 422 (26.0) |  |
| **Macroscopic vascular invasion** |  |  |  |  | <0.001 |  |  |  | <0.001 |  |  |  | <0.001 |  |  | 0.033 |  |  |  | 0.007 |  |  | 0.104 |  |  | <0.001 |  |  | <0.001 |
| Present | 416  (25.6) | 148  (9.1) | 220  (13.5) | 48  (3.0) |  | 158  (9.7) | 210  (12.9) | 48  (3.0) |  | 62  (3.8) | 299  (18.4) | 55  (3.4) |  | 359  (22.1) | 57  (3.5) |  | 283  (17.4) | 122  (7.5) | 11  (0.7) |  | 288  (17.7) | 128  (7.9) |  | 116  (7.1) | 300  (18.5) |  | 160 (9.8) | 256 (15.8) |  |
| Absent | 1209  (74.4) | 677  (41.7) | 442  (27.2) | 90  (5.5) |  | 738  (45.4) | 381  (23.4) | 90  (5.5) |  | 436  (26.8) | 643  (39.6) | 130  (8.0) |  | 1089  (67.0) | 120  (7.4) |  | 908  (55.9) | 263  (16.2) | 38  (2.3) |  | 887  (54.6) | 322  (19.8) |  | 565  (34.8) | 644  (39.6) |  | 745 (45.8) | 464  (28.6) |  |
| **Extrahepatic metastasis** |  |  |  |  | <0.001 |  |  |  | <0.001 |  |  |  | <0.001 |  |  | 0.025 |  |  |  | <0.001 |  |  | 0.042 |  |  | <0.001 |  |  | <0.001 |
| Present | 192  (11.8) | 67  (4.1) | 100  (6.2) | 25  (1.5) |  | 71  (4.4) | 96  (5.9) | 25  (1.5) |  | 22  (1.4) | 142  (89.2) | 28  (1.7) |  | 162  (10.0) | 30  (1.8) |  | 117  (7.2) | 66  (4.1) | 9  (0.6) |  | 127  (7.8) | 65  (4.0) |  | 51  (3.1) | 141  (8.7) |  | 69 (4.2) | 123  (7.6) |  |
| Absent | 1433  (88.2) | 758 (46.6) | 562  (34.6) | 113  (7.0) |  | 825  (50.8) | 495  (30.5) | 113  (7.0) |  | 476  (29.3) | 800  (49.2) | 157  (9.7) |  | 1286  (79.1) | 147  (9.0) |  | 1074  (66.1) | 319  (19.6) | 40  (2.5) |  | 1048  (64.5) | 385  (23.7) |  | 630  (38.8) | 803  (49.4) |  | 836 (51.4) | 597 (36.7) |  |
| **BCLC stage** |  |  |  |  | <0.001 |  |  |  | <0.001 |  |  |  | <0.001 |  |  | 0.018 |  |  |  | 0.001 |  |  | 0.037 |  |  | <0.001 |  |  | <0.001 |
| A | 468  (28.8) | 285  (17.5) | 141  (8.7) | 42  (2.6) |  | 306  (18.8) | 120  (7.4) | 42  (2.6) |  | 198  (12.2) | 216  (13.3) | 54  (3.3) |  | 415  (25.5) | 53  (3.3) |  | 347  (21.4) | 109  (6.7) | 12  (0.7) |  | 333  (20.5) | 135  (8.3) |  | 231  (14.2) | 237  (14.6) |  | 299 (18.4) | 169 (10.4) |  |
| B | 642  (39.5) | 352 (21.7) | 256  (15.8) | 34  (2.1) |  | 390  (24.0) | 218  (13.4) | 34  (2.1) |  | 255  (13.8) | 357  (22.0) | 60  (3.7) |  | 588  (36.2) | 54  (3.3) |  | 500  (30.8) | 122  (7.5) | 20  (1.2) |  | 486  (29.9) | 156  (9.6) |  | 302  (18.6) | 340  (20.9) |  | 405 (24.9) | 237 (14.6) |  |
| C | 515  (31.7) | 188  (11.6) | 265  (16.3) | 62  (3.8) |  | 200  (12.3) | 253  (15.6) | 62  (3.8) |  | 75  (4.6) | 369  (22.7) | 71  (4.4) |  | 445  (27.4) | 70  (4.3) |  | 344  (21.2) | 154  (9.5) | 17  (1.0) |  | 356  (21.9) | 159  (9.8) |  | 148  (9.1) | 367  (22.6) |  | 201 (12.4) | 314 (19.3) |  |

Variables are expressed as no. (%).

Abbreviation: HBV, hepatitis B virus; HCV, hepatitis C virus; ALBI grade, Albumin-bilirubin grade; BCLC stage, Barcelona Clinic Liver Cancer stage; GPS, Glasgow Prognostic Score; mGPS, modified Glasgow Prognostic Score; Hs-mGPS, high-sensitivity modified Glasgow Prognostic Score; NLR, neutrophil-to-lymphocyte ratio; PLR, platelet-to-lymphocyte ratio; PNI, Prognostic Nutritional Index; SII, systemic immune-inflammation index; LCR, Lymphocyte-C-reactive protein ratio.
